# Concurrent Reflectance Confocal Microscopy and Laser Doppler Flowmetry to Improve Skin Cancer Imaging: A Monte Carlo Model and Experimental Validation

**DOI:** 10.3390/s16091411

**Published:** 2016-09-01

**Authors:** Alireza Mowla, Thomas Taimre, Yah Leng Lim, Karl Bertling, Stephen J. Wilson, Tarl W. Prow, H. Peter Soyer, Aleksandar D. Rakić

**Affiliations:** 1 School of Information Technology and Electrical Engineering, The University of Queensland, St. Lucia, Brisbane 4072, Australia; a.mowla@uq.edu.au (A.M.); ylim@itee.uq.edu.au (Y.L.L.); bertling@itee.uq.edu.au (K.B.); wilson@itee.uq.edu.au (S.J.W.); 2 School of Mathematics and Physics, The University of Queensland, St. Lucia, Brisbane 4072, Australia; t.taimre@uq.edu.au; 3 Dermatology Research Centre, The University of Queensland, School of Medicine, Translational Research Institute, Brisbane 4102, Australia; t.prow@uq.edu.au (T.W.P.); p.soyer@uq.edu.au (H.P.S.)

**Keywords:** skin cancer detection, Monte Carlo modelling, laser feedback interferometry, reflectance confocal microscopy, laser Doppler flowmetry

## Abstract

Optical interrogation of suspicious skin lesions is standard care in the management of skin cancer worldwide. Morphological and functional markers of malignancy are often combined to improve expert human diagnostic power. We propose the evaluation of the combination of two independent optical biomarkers of skin tumours concurrently. The morphological modality of reflectance confocal microscopy (RCM) is combined with the functional modality of laser Doppler flowmetry, which is capable of quantifying tissue perfusion. To realize the idea, we propose laser feedback interferometry as an implementation of RCM, which is able to detect the Doppler signal in addition to the confocal reflectance signal. Based on the proposed technique, we study numerical models of skin tissue incorporating two optical biomarkers of malignancy: (i) abnormal red blood cell velocities and concentrations and (ii) anomalous optical properties manifested through tissue confocal reflectance, using Monte Carlo simulation. We also conduct a laboratory experiment on a microfluidic channel containing a dynamic turbid medium, to validate the efficacy of the technique. We quantify the performance of the technique by examining a signal to background ratio (SBR) in both the numerical and experimental models, and it is shown that both simulated and experimental SBRs improve consistently using this technique. This work indicates the feasibility of an optical instrument, which may have a role in enhanced imaging of skin malignancies.

## 1. Introduction

Melanoma and keratinocyte skin cancers (KSCs) are the most common types of cancers affecting fair-skinned populations [1,2,3] with incidence increasing about 4% per year worldwide [4]. Although the cure rate is high in KSCs, such as basal cell carcinoma (BCC) and squamous cell carcinoma (SCC), they can become aggressive [3,4,5]. Squamous cell carcinoma, responsible for about 20% of KSCs, is associated with moderate risk of metastasis [3,4]. Basal Cell Carcinoma (BCC) constitutes approximately 80% of KSCs, and although metastases are rare, BCC still imposes a significant burden of disease [3,4]. To alleviate this burden, early non-invasive detection schemes are required.

Most of the technical developments in non-invasive skin cancer detection are based on imaging reflected light from the skin surface or deeper tissues and examination of the image by an expert clinician. These images are interpreted on the basis of structural or functional changes manifest in the optical properties of skin tissue due to neoplastic changes. Changes in optical properties (scattering and absorption coefficients) of neoplastic tissue are due to morphological, molecular and ultrastructural changes [6]. Optical properties of normal skin have been extensively studied [7], and a comparison between healthy and neoplastic skin lesions has been studied in other work [8]. Diffuse reflectance spectroscopy was used to study the light reflectance from tumourous tissues [9,10]. Furthermore, reflectance confocal microscopy (RCM), which can be used in reflectance mode for in vivo applications [11,12], evolved to be one of the most promising skin cancer detection tools [13,14,15,16,17]. RCM increases the contrast of a microscope image, provided from within a specimen, by restricting the observed volume.

Other biological traits can be used in early detection of cancers. One of the hallmarks of cancer is angiogenesis [18], as vasculogenic tumour cells cause neovascularization, which plays an important role in tumour growth and cancer metastasis [19,20]. Angiogenesis occurs very early in the multi-stage development of invasive cancers, and it can be attributed to the microscopic premalignant phase of neoplastic progression [18]. Therefore, using a technique such as laser Doppler flowmetry (LDF), a well-established blood perfusion measurement technique [21], to map skin blood perfusion in benign and malignant skin tumours, has potential as a tool in early skin cancer detection [22,23,24]. LDF has previously been used for cancer detection purposes and in diagnosing other diseases. Stücker et al. investigated the possibility of differentiating between benign and malignant skin tumours by assessing the level of microvascularization using laser Doppler perfusion imaging [23,24,25]. Saravanamuthu et al. used laser Doppler perfusion imaging of vulval skin blood flow to demonstrate abnormal perfusion in vulval cancer [26]. Seifalian et al. examined breast skin perfusion in cases of underlying breast cancer with the hypothesis that increased regional metabolism will result in higher perfusion. It was shown that breast cancer causes higher perfusion in breast skin [27]. This modality suffers from limitations, such as low specificity, because other peripheral vascular diseases, such as diabetes, arteriosclerosis and vasospastic conditions, can cause similar disturbances in the skin perfusion [28].

In brief, all of the aforementioned techniques suggest that optical biomarkers of malignant changes in skin, used for detection purposes, can be roughly separated into two groups: one related to morphological changes in the skin tissue (quantified through techniques, such as RCM) and the other related to functional changes, such as blood flow (quantified through techniques, such as LDF). The purpose of this work is to combine these two modalities to improve the efficacy of the imaging system. The combination of multiple imaging modalities to improve imaging efficacy has been explored previously in the literature. Chen et al. proposed a method that combines the optical coherence tomography and LDF to measure the blood perfusion and tissue structure at the same time [29]. Yaroslavsky et al. combined multispectral polarized light imaging and confocal microscopy to localize KSCs more accurately [30].

Our approach to combine RCM and LDF is to employ a special configuration for RCM, which is able to pick up the Doppler shift in the frequency of the laser beam. We propose to use a laser feedback interferometry (LFI) system for this purpose. This technique is based on interferometry, which is one of the methods that can be used to extract the Doppler signal. By using LFI in a confocal configuration using a vertical-cavity surface-emitting laser (VCSEL), both confocal reflectance and Doppler flowmetry signals can be acquired concurrently in the same scan without changing the hardware. In the system, the aperture of the VCSEL acts as an RCM pinhole and defines the sensing volume (the lateral resolution and the depth of field of the instrument). The back-reflected beam then interferes with the intra-cavity field to give rise to the Doppler signal. An LFI system is a compact sensing tool which is based on the self-mixing effect [31,32,33,34,35]. It has been previously used for confocal reflectance [36,37] and Doppler flowmetry [38,39] imaging purposes.

We examine the proposed technique numerically by applying it to typical simulated models of KSCs, carried out by means of Monte Carlo algorithms specifically adapted for this application, and experimentally by conducting an experiment using an LFI system providing in-depth images of a dynamic turbid medium flowing in a microfluidic channel. We define a signal to background ratio (SBR) parameter to quantify the improvement in the contrast of an image resulting from the multiplication of the RCM and LDF images. There is consistency between simulated and experimental results, and it is shown that SBR for combination images improves significantly in both simulated and experimental studies. The common sensing platform for both measures being LFI, provides an imaging technique where both the confocal reflectance and Doppler flowmetry images can be acquired concurrently during one single scan. Such an image can be used to discriminate between changes in the blood content and optical properties of skin tumours.

The paper is structured as follows: We discuss the methodology in Section 2, describing confocal LFI, LDF and Monte Carlo simulations in its subsections. Section 3 presents the results of the simulated skin tumour models. Section 4 and its subsections describe the experimental setup, technical considerations and results. This section is followed by the discussion in Section 5, and finally, we give the conclusion.

## 2. Methodology

Our approach to study the technique is through Monte Carlo simulation of photon-tissue interactions and experimental validation by examining a microfluidic channel containing a dynamic turbid medium. Our numerical configuration consists of tumourous skin models and a confocal LFI optical system [32,34,38,40,41]. We described skin using a six-layer model, and tumourous regions were included as areas with enhanced red blood cell (RBC) activity and altered optical properties. We considered two typical models for skin tumours, and the characteristics of these typical types of skin cancer were defined based on the reported values in the literature. To acquire the signal and form the image in simulations, we raster scanned the confocal LFI model over the skin model. The signal was then processed to extract confocal reflectance (from the number of detected photons) and the Doppler signal (from the Doppler power spectrum). Then, the confocal reflectance and Doppler images were combined by multiplying the signals point by point. Among the different techniques to combine the images, we chose multiplication, as it suppresses the background noise and improves the SBR. The combined image provided by this technique is affected by four different parameters: (i) RBC concentration; (ii) RBC velocities; (iii) tissue scattering coefficient (iv) and tissue absorption coefficient; all of which are optical biomarkers of neoplastic changes. Following the numerical part, we include an experimental part in the paper to evaluate the technique. Experiments were conducted using a confocal LFI system imaging a microfluidic channel containing a dynamic turbid medium, consisting of a diluted Intralipid (20%) with deionized-water. We obtained the confocal reflectance and Doppler images and combined them to make the multiplication image. There is a high level of concordance between the experimental and numerical results, which validates the technique.

In the following subsections, we discuss confocal LFI, LDF and also the specifications of our specifically-adapted Monte Carlo algorithm to examine the multi-layer tumourous skin model.

### 2.1. Confocal Laser Feedback Interferometry

**Figure 1:**
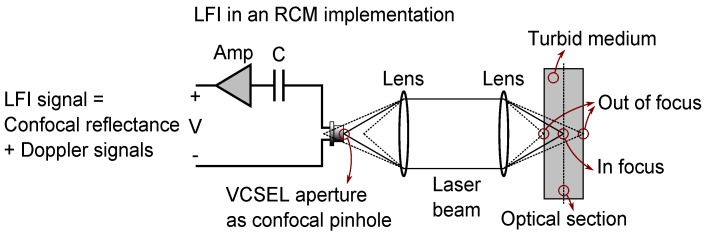
LFI with an RCM implementation based on a VCSEL as both the emitter and the detector. The VCSEL aperture with a diameter of a few microns acts as the spatial RCM pinhole in this structure, and the LFI signal is generated due to the self-mixing effect inside the VCSEL cavity.

One of the most widely-accepted non-invasive modalities used to provide in vivo images inside living organisms is RCM [42]. The idea of RCM is to eliminate out of focus light by situating a spatial pinhole at the focal point of the lens and to increase the resolution and contrast of the image in addition to the ability to provide in vivo images in the reflection mode. The idea was first proposed in 1957 [43], but it was only after the invention of lasers and computer systems that it quickly found its way in the biomedical community [44] and, recently, in applications, such as skin cancer detection [13]. Depending on the RCM configuration, the specimen is illuminated in a different way. A limited portion of light coming from an infinitesimal spatial sensing volume, which is able to pass through the lensing system and spatial pinhole, is detected in the system to form the image. To perform optical sectioning in an in vivo application, the light and filtering system should be scanned over the specimen. A confocal LFI system using a semiconductor laser, such as a VCSEL, can be interpreted as a peculiar embodiment of an RCM system [36,37,45,46,47,48]. In a confocal LFI system, which works based on the self-mixing effect [32,34], light emitted from the laser is reflected off a distant target. A small potion of the beam that is reflected back from a very small sensing volume, in a focused beam configuration with a high numerical aperture beam, is able to pass through the optical system and VCSEL’s aperture to re-enter the laser cavity. The interference of the reflected and intra-cavity field inside VCSEL’s cavity gives rise to a range of perturbations in the operation of the laser, which can be detected by monitoring the laser’s output optical power or terminal voltage for sensing purposes. The diameter of the aperture of a typical VCSEL is in the range of a few micrometers up to about 10 micrometers [49], which can be considered as the equivalent of an RCM pinhole in a confocal LFI configuration. Using a high numerical aperture (NA) objective lens is required to have a very small sensing volume, and a laser at a near-infrared optical window guarantees the maximum penetration depth in biological tissues. Figure 1 depicts the schematic of a confocal LFI system in an RCM implementation. The detection method in such a peculiar RCM is based on interferometry rather than photomultiplier tubes or avalanche photodiodes in conventional RCMs, and this feature can be exploited to extract the Doppler shift from the signal. The Doppler shift in the frequency of the beam due to the movement of the red blood cells in a living tissue is in the range of a few kilohertz, which is impossible to detect without an interferometric scheme. In Section 4, further explanations are included about how to extract both confocal reflectance and Doppler signals in an experimental situation.

### 2.2. Laser Doppler Flowmetry

**Figure 2:**
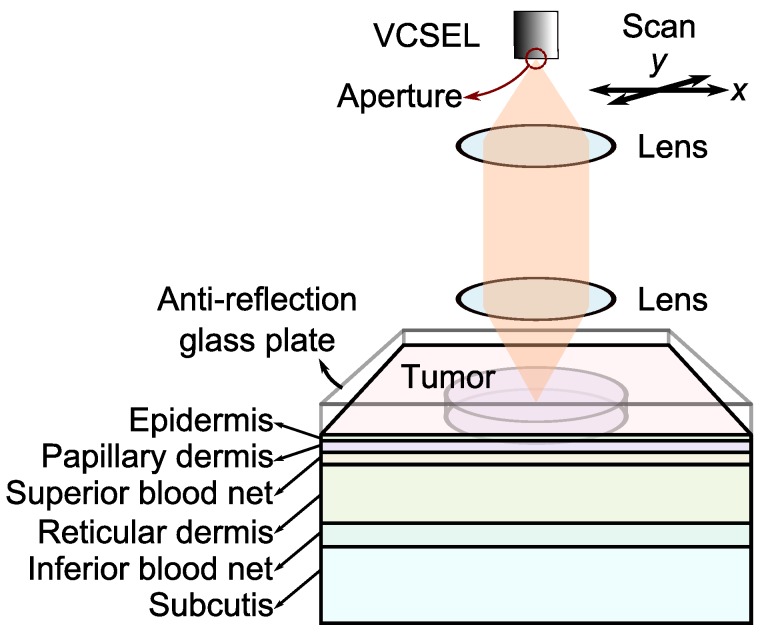
Block diagram of numerical model including a confocal LDF system raster scanning a six-layer skin tissue model to make the confocal reflectance and Doppler images of an embedded tumour model.

Laser Doppler flowmetry has been extensively used in microcirculatory blood flow measurements, as it is a non-invasive method. In laser Doppler perfusion imaging, a collimated laser beam is raster scanned on the skin area of interest to create the perfusion map [28]. The operation of an LDF system is based on the Doppler shift in the frequency of the coherent beam. Photons at wavelengths with a long penetration depth enter the skin tissue and collide with scatterers and chromophores. They will either be scattered or absorbed. The photons that are scattered from moving scatterers, such as RBCs, undergo Doppler shifts. Doppler shift is calculated from the following Equation [50]:

(1) $$\Delta f=\frac{1}{2\pi }\left(k_{s}-k_{i}\right)\cdot v\;,$$

where Δf is the Doppler shift in the photon’s frequency, $k_{s}$ and $k_{i}$ are the wave vectors of the scattered and incident waves, respectively, and v is the velocity vector of the moving scatterer. From the diffused photons in the turbid skin medium, some of them make their way out of the tissue and return to the detection system of the LDF, which usually involves an interferometric technique and a photodetector to acquire the beat frequency. Perfusion is defined as the product of the average RBCs’ velocity and concentration and is proportional to the first moment of the power spectrum of the detected signal [51]:

(2) $$\text{Perf}\propto \int_{0}^{\infty }\omega P\left(\omega \right)d\omega \;,$$

where *ω* is the angular frequency and P(ω) is the Doppler power spectrum. By raster scanning the beam on the skin surface, we can create the perfusion map. In our numerical model, we perform this scan by moving the model of the confocal LFI system over the skin model. The schematic of the numerical model is depicted in Figure 2. As our numerical model shows, detailed in Section 2.3, light is emitted from a simulated 850-nm laser diode and focused on the six-layer skin tissue model. An anti-reflection glass layer is assumed on the top of skin model to reduce specular reflection. A portion of the photons enter the turbid medium of the tissue. Photons will diffuse after interacting with chromophores and scatterers. A portion of the diffused light makes its way out of the tissue and re-enters the laser cavity. Doppler shifted photons, which re-enter the laser cavity, contribute to the Doppler power spectrum. Photon frequencies shift due to collisions with moving RBCs. At each point, we numerically calculated the confocal reflectance signal by counting the number of detected photons, which make their way back into the laser cavity through the optical system and the aperture of the VCSEL; Figure 2. At the same point, the Doppler power spectrum is defined as the histogram of the detected photons’ Doppler frequency shifts, and perfusion is the first moment of the optical Doppler spectrum calculated from Equation (2), which we defined as the Doppler signal in this paper.

In the proposed technique, we can neglect technical difficulties, such as measuring the blood perfusion in absolute value and distinguishing between the contributions of RBC’s concentration and velocities to the Doppler signal, which commonly exist in LDF systems [21]. That is because, we are examining the relative values coming from the lesion compared to the surrounding normal area, and there is no need to distinguish between the concentration and velocity of RBCs, as both of them exist in the tumourous neovascularization and both are indicators of cancer growth.

### 2.3. Monte Carlo Simulation

Monte Carlo is the gold standard in the study of photon-tissue interaction and has been used extensively in the field [52,53,54]. In our numerical model, at each scanning site, photons are projected toward the skin tissue, numerically, in a hyperboloid shape to resemble the focused Gaussian beam [55]. We separately executed the simulation for confocal reflectance and Doppler signals with 0.32 and 1.6 million photons, respectively. We defined numerical lenses in the model based on the physical characteristics of commercially available Thorlabs C240TME-B lenses with both a clear aperture diameter and an effective focal length of 8 mm. The features of the numerical lens determine the shape of the focused beam. We assumed the beam diameter at the objective lens is 1.96 mm. This beam diameter along with a 0.5 numerical aperture and 8 mm focal length of the numerical lens result in a beam spot diameter of about 4 µm at the focal point. We employed a variance reduction technique when calculating the Doppler signal, in which photons with large Doppler shifts can split into up to 3200 photons [56]. We assumed the wavelength of the photons to be 850 nm, as the water absorption coefficient is almost negligible at this wavelength [57]. As shown in Figure 2, we used a six-layer skin model assuming an anti-reflective coating model on the top to avoid specular reflection. We adopted the skin model proposed in the paper of Fredriksson et al., which was validated through their experiments [56]. We assumed the refractive index of the glass plate was close to the skin refractive index to have the index matching. The names and characteristics of the skin layers are listed in Table 1. We approximated all of the layers’ refractive indices to be 1.4 to avoid index mismatching between the layers [56]. We defined three types of blood components with average velocities of 0.3, 3.0 and 30 mm/s, each present in different volumes within the layers [56]. These low, middle and high velocity blood components can be associated with capillaries, venules (small veins and arterioles) and larger blood vessels. We assumed random RBC directions with a uniform distribution in 3D space. To approximate laminar blood flow, we defined the RBC velocities in a uniform random distribution from zero to two-times the average velocity components [56]. Melanin, as the main absorber of light, exists only in the epidermis layer of healthy skin tissue. Epidermis is the only layer with no blood components. We defined initial absorption and scattering coefficients for each layer and then added the optical properties of melanin and blood components in each layer [56], as listed in the Table 1. Blood has absorption and scattering coefficients of 0.5 and 222 mm^−1^, respectively, and melanin has an absorption coefficient of 15 mm^−1^ and no scattering coefficient. The phase function of a scatterer determines the scattering direction of the photons. Here, all of the non-blood scatterers have a Henyey–Greenstein phase function with an anisotropy factor of 0.85, and RBCs have a Gegenbauer kernel phase function [58] with $\alpha_{\text{Gk}}=1.0$ and $g_{\text{Gk}}=0.948$, resulting in an anisotropy factor of 0.991 [56]. We used a variance reduction technique to focus on the Doppler shifted photons [56].

We numerically raster scanned an area of 10 × 10 mm^2^ on the skin tissue model with 30 steps at each side and created 30 × 30 pixel images. We discussed the photon-tissue interactions in Section 2.2, and the configuration can be seen in Figure 2. The detected photon population (which determines the confocal reflectance) is the portion of photons that exit from the skin model, pass through the lenses and VCSEL’s aperture and return back to the laser cavity. To speed up the numerical calculations, we approximated the radius of the aperture to be 45 µm. Photons move through the lenses based on the ray tracing rules, and the geometry of lens surfaces is derived from the data for C240TME-B.

## 3. Simulation Setup and Results

### 3.1. Setup

Tumorous regions in the numerical model are roughly defined as thin cylindrical volumes with diameters of 5 mm and thicknesses of 0.3 mm, laying within the second and third layers of papillary dermis and superior blood net, situated at a depth of 0.075 to 0.375 mm, as illustrated in Figure 2. To form the images, we configured the numerical model to keep the focal point of the beam in the middle of the tumour model at the depth of 0.225 mm from the surface. To study the applicability of the technique, we applied it numerically to two typical types of KSC and called them mild KSC (KSC1) and severe KSC (KSC2). Tumorous abnormalities for these typical types of skin cancer are defined in the simulation as higher RBC concentration and velocities and lower absorption and scattering coefficients.

Stücker et al. used a commercial laser Doppler perfusion imaging device to measure perfusion in different types of skin cancer [23,24]. They reported increases in the perfusion of BCCs and melanocytic nevi with respect to average healthy skin by factors of approximately 1.8 and 3.8, respectively [23]. In our numerical model, we defined the RBC concentrations of two- and three-times higher than healthy skin for typical KSC1 and KSC2 and RBC velocities of 1.5-times higher than healthy skin for both types. These abnormalities resulted in increases in the perfusion of KSC1 and KSC2 of about 1.7 and 3.7 higher than healthy skin, which we took to be roughly compatible with the data reported in [23] and acceptable for our purposes. On the other hand, Salomatina et al. measured the in vitro optical properties of infiltrative BCC, nodular BCC and invasive SCC in a large spectral range [8]. Their results show remarkable drops in the scattering and absorption coefficients of the tissues affected by these types of KSCs at some wavelengths. For instance, at 850 nm, the scattering and absorption coefficients of nodular BCC and SCC, reduce by factors of about 0.6, all with respect to normal dermis tissue [8]. However, these results are from in vitro measurements, and to apply them to an in vivo model, the effect of blood should be included. These numbers can be quite different for different types of skin cancer. In our numerical model, we assumed the scattering and absorption coefficients to reduce by factors of 0.6 with respect to normal dermis skin tissue for both typical types of KSC1 and KSC2.

To use ex vivo measurement results of tissue optical properties in an in vivo numerical model, we need to add the effect of average blood contents of the tissue to the measured ex vivo optical properties using the following expression [59]:

(3) $$\mu_{\text{in vivo}}\left(\lambda \right)=V_{\text{blood}}\times \mu_{b}\left(\lambda \right)+\left(1-V_{\text{blood}}\right)\times \mu_{\text{ex vivo}}\left(\lambda \right)\;,$$

where $\mu_{\text{in vivo}}$ and $\mu_{\text{ex vivo}}$ are the in vivo and ex vivo optical properties at the wavelength *λ*, respectively, $V_{\text{blood}}$ is the volume fraction of blood in the tissue and $\mu_{b}$ is the optical property of blood. We mentioned optical properties of blood and bloodless tissue in Section 2.3. The typical average value for the blood volume fraction in the skin layers is about 0.2%, although its value is about 2%–5% in the venous plexus [60]. In our numerical model, we used the blood volume fractions given in Table 1.

### 3.2. Results

**Figure 3:**
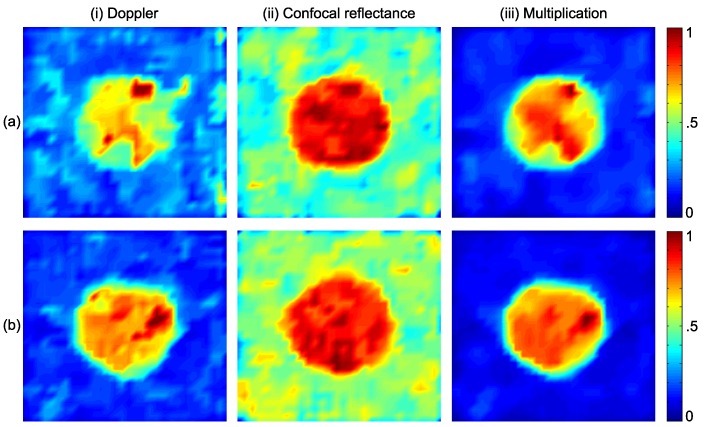
Monte Carlo simulated images of typical (**a**) KSC1 and (**b**) KSC2. Parts (i) and (ii) of the images depict blood Doppler and confocal reflectance images of 5 mm diameter tumours, respectively. Part (iii) shows the multiplication image, which is formed by multiplying Parts (i) and (ii), pixel-by-pixel.

Parts (i) and (ii) of Figure 3a show the simulated Doppler and confocal reflectance images for the typical KSC1, respectively. Furthermore, Parts (i) and (ii) of Figure 3b show the simulated Doppler and confocal reflectance images for the typical KSC2, respectively. Part (iii) of Figure 3a,b shows the multiplication images. Results are normalized to the peak Doppler and confocal reflectance values, and it can be seen that increasing RBCs’ concentration results in higher contrast by comparing Part (i) of Figure 3a,b. We filtered all of the images to eliminate spikes using two-dimensional median filtering in MATLAB (R2015a, UQ University License, Brisbane, Australia), and all of the values are relative and normalized to arbitrary units.

In order to quantify the numerical results, we defined an SBR parameter as the ratio of the average signal from the tumourous area to the average signal from the normal surrounding skin area obtained from the simulations. This parameter can be formulated as follows:

(4) $$SBR=\frac{\frac{1}{N_{T}}\sum_{i\in T}Sig_{i}}{\frac{1}{N_{\text{BG}}}\sum_{i\in BG}Sig_{i}}\;,$$

where $N_{T}$ and $N_{BG}$ are the number of pixels in the tumourous and background areas, respectively, and $Sig_{i}$ is the signal value at pixel *i*. Furthermore, *T* and *BG* denote the set of pixel indices associated with the tumourous and background areas, respectively. In the case of KSC1 shown in Part (a) of Figure 3, the SBR for Doppler, confocal reflectance and multiplication images is 1.4, 1.3 and 2.8, respectively. In the case of KSC2 shown in Part (b) of Figure 3, the SBR for Doppler, confocal reflectance and multiplication images is 2.9, 1.2 and 5.0, respectively. As can be inferred from these numbers, the combination of the two techniques is enhancing the SBRs of the images dramatically. The minimum increase factor is about 1.7 in the case of KSC2, which is the ratio of the SBR of the multiplication image to its value for the Doppler image, and in all of the other cases SBRs increase by factors greater than 1.7.

**Figure 4:**
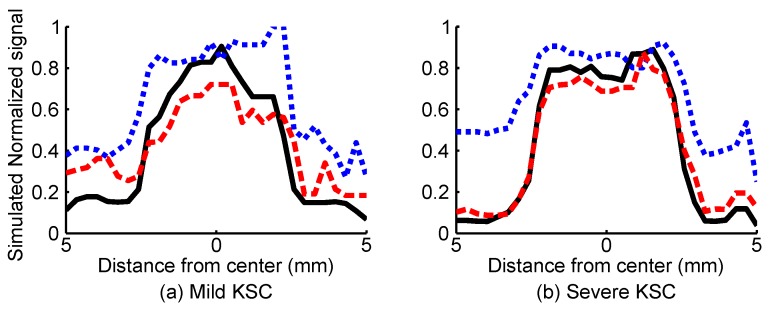
Improvement in the signal to background ratio as a result of multiplying Doppler flowmetry and confocal reflectance images. Red broken, blue dotted and black solid lines in (**a**) and (**b**) of this image correspond to the Doppler flowmetry, confocal reflectance and multiplication signals acquired along center-crossing horizontal axes in (a) and (b) of Figure 3, which correspond to typical KSC1 and KSC2 simulated models, respectively.

To more clearly see the SBR improvement, we plotted the signals along the center-crossing horizontal axis in Parts (a) and (b) of Figure 3 and present these plots in Figure 4. Parts (a) and (b) of Figure 4 correspond to the simulated models of KSC1 and KSC2, respectively.

## 4. Experimental Validation

To examine the feasibility of an experimental device for concurrent RCM and LDF, which is applicable for biomedical imaging purposes, we used a confocal LFI system to image a dynamic turbid medium [61]. The purpose of this experiment is to provide two independent images of confocal reflectance and Doppler flowmetry as a result of a single scan. Images should be able to depict the morphological features and flow characteristics of a dynamic turbid medium at a depth below the surface (where the confocal plane is situated), which is penetrable to the laser beam, and with respect to a substrate. As we discussed briefly in Subsection 2.1, confocal LFI can be considered as a particular embodiment of RCM, which possesses an interferometric nature. It has been used previously to monitor blood perfusion [62] and also in confocal reflectance [36,37,48] and Doppler flowmetry [38] imaging systems.

**Figure 5:**
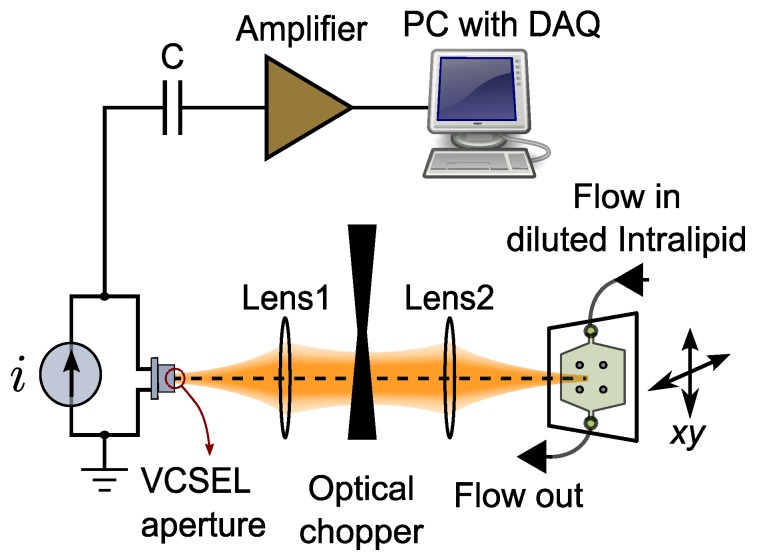
Experimental confocal LFI apparatus in the dual-modality imaging application, scanning a microfluidic channel containing diluted Intralipid flow.

Figure 5 shows a schematic of the experimental apparatus we constructed for this purpose. As can be seen and compared in Figure 1, Figure 2 and Figure 5, the suggested experimental setup is based on the concepts of the technique and compatible with the optical system in the numerical model.

### 4.1. Experimental Setup

**Figure 6:**
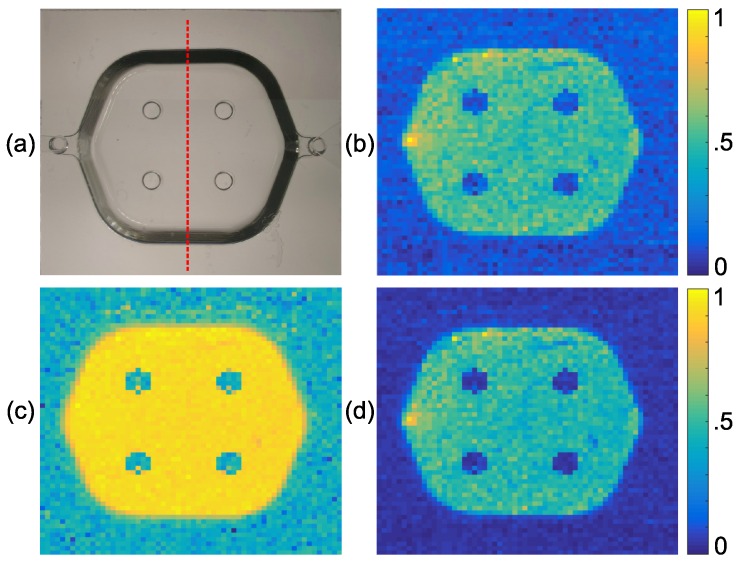
(**a**) Top view of the microfluidic channel (the red broken line shows the center-crossing axis alone which SBR was calculated) and experimental normalized (**b**) Doppler flowmetry, (**c**) confocal reflectance and (**d**) multiplication images.

As illustrated in Figure 5, an 850-nm VCSEL operating at 3.55 mA was used in this setup. The beam divergence angle of the VCSEL was about seven degrees, and its temperature was controlled at 35 °C to get an optimum signal [63]. The beam was collimated using a lens with a focal length and numerical aperture of 8 mm and 0.5, respectively (Model C240TME-B, Thorlabs, Inc.; Lens 1 in Figure 5), and focused using a second lens with a focal length and numerical aperture of 3.1 mm and 0.68, respectively (Model C330TME-B, Thorlabs, Inc.; Lens 2 in Figure 5). The second lens had a focal beam spot diameter and Rayleigh length of about 1.8 and 2.9 µm, respectively, which was appropriate for microscopy applications. We need to apply the technique to a dynamic turbid medium, which is a semi-transparent medium, including both dynamic and static scatterers and absorbers, with a scattering coefficient well above the absorption coefficient. Such a target provides variations in both optical properties and flow distribution, which fits our purpose of dual-modality imaging. Therefore, we chose to use diluted Intralipid flow in a microfluidic channel as an imaging sample. Intralipid is a fat emulsion used as an intravenous nutrient, and its optical properties have been studied before [64]. It has also been used numerously as a phantom for biomedical tissues [65]. We diluted Intralipid 20% with deionized-water with a ratio of 30:1 and pushed it through a microfluidic channel with a channel area of about 8 × 10 mm^2^ and a channel thickness of about 1 mm. The top view of this microfluidic channel can be seen in Figure 6a, and also, it is shown in Figure 5 as the target. A syringe pump was used to push the mixture with a flow rate of 800 µL/min through the channel. The laser beam was focused at a depth of about 500 µm below the surface of the diluted Intralipid, and the focal plane was kept at this depth throughout the scan. In order to make the images, we used two motorized translation stages (Zaber Technologies Inc.) to move the microfluidic channel vertically and horizontally, to raster scan the laser beam over the channel. An area of 12 × 12 mm^2^ in 61 × 61 steps, at a 200-µm pitch, was scanned. In the proposed set up, the laser beam is perpendicular to the target, which is different from conventional LDF systems. This geometry is more common in biomedical imaging applications, and the Doppler power spectrum, which is a broadened signal around the frequency of zero, was extracted from the focused part of the beam with a high numerical aperture. At each step of the scan, the LFI signal was acquired from the VCSEL junction voltage and was fed into a 16-bit DAQ card after amplification. The time domain voltage signal was sampled at a rate of 60 kS/s, and 3 kS was used as the signal time sequence, which corresponds to 50 ms. To convert the time domain signal into the frequency domain, we applied fast Fourier transform to the time sequence and used a Savitzky–Golay filter in MATLAB (R2015a, UQ University License, Brisbane, Australia) to filter out the frequency domain signal.

Because semiconductor lasers are noisy at DC or low frequencies [63], we AC-coupled the VCSEL junction voltage when amplifying it. Therefore, we needed to modulate the laser beam to extract the confocal reflectance signal. As can be see in Figure 5, we used an optical chopper to modulate the beam at the frequency of 1 kHz. Modulating the beam created two different levels of feedback, which was then detected and used to quantify the level of reflection [66].

### 4.2. Experimental Results

**Figure 7:**
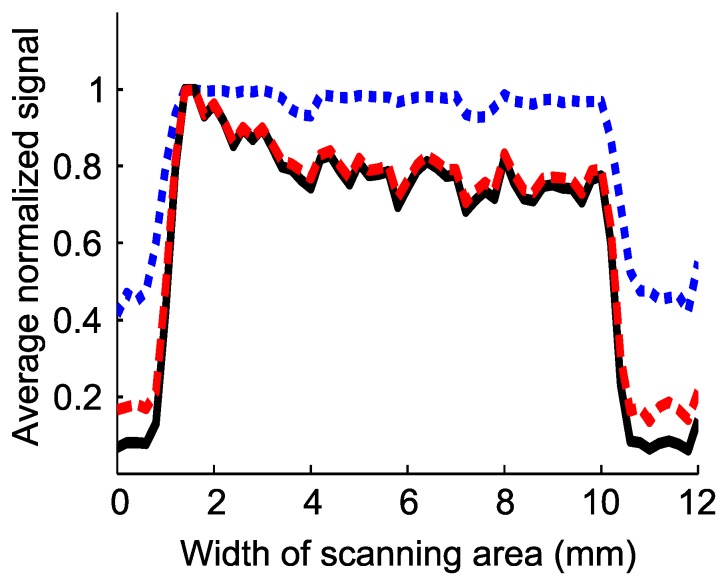
Red broken, blue dotted and black solid lines show average Doppler flowmetry, confocal reflectance and multiplication signals, acquired along the center-crossing axis of the microfluidic channel (shown with the red broken line in Figure 6a), respectively.

Scan results are illustrated in Figure 6. Doppler flowmetry, confocal reflectance and multiplication images are shown in Figure 6b–d, respectively. There are four pillars situated in the middle of the microfluidic channel by the manufacturer for stability purposes, which can be seen as four dots at the noise level in all of the images in Figure 6. Doppler flow distribution, depicted in (b), has the highest flow rate at the input of the channel on the left side of it. Confocal reflectance distribution, depicted in (c), has an even distribution all over the channel, as expected, because the optical properties of the turbid medium are maintained constant throughout the volume. The background noise level (signal from the outside of the channel area) is higher in (c) compared to (b), as there is a small amount of reflection from the surface of the microfluidic channel, although the surface is not at the focal plane, which is higher than the background noise in (b) (which is the overall rise in the Doppler spectrum in the absence of flow due to noises). The multiplication image, (d) of Figure 6, is made by multiplying (b) and (c), pixel-by-pixel. It can be noticed from (d) that the background noise is reduced in the multiplication image. Similar to what we did in the case of the numerical model in Section 3, we quantified the improvement in the contrast of the multiplication image by defining an SBR parameter similar to the way we defined it in Equation 4, as the average level of the signal from the flow area to the average level of the signal outside the flow area, along a center-crossing axis shown by a red broken line in Figure 6a. Average Doppler flowmetry, confocal reflectance and multiplication signals, acquired along the red broken line in Figure 6a, are shown in Figure 7. It is clear from this image that multiplication signal (black solid line) has a lover noise level (at left and right wings) compared to the other two signals. SBR for Doppler flowmetry, confocal reflectance and multiplication signals is 4.1, 1.9 and 7.2, respectively. SBR of the multiplication signal is improved by factors of 1.8 and 3.7 with respect to Doppler flowmetry and confocal reflectance signals, respectively. Therefore, the minimum improvement factor for SBR in the experimental result is 1.8, which is consistent with the figure for the numerical model, which was 1.7. This shows good agreement between experimental and numerical results.

**Figure 8:**
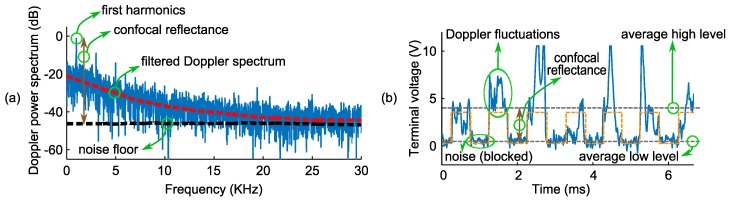
(**a**) Frequency and (**b**) time domain confocal LFI signals, obtained from the voltage of the VCSEL terminals. Red and black broken lines in (**a**) show the filtered Doppler spectrum and noise floor (in the absence of target), and the first large spike in (**a**) is the first harmonic of the signal (as the beam is modulated at 1 kHz); its magnitude with respect to noise floor is a measure of the confocal reflectance signal. The orange broken line in (**b**) shows the modulating signal at 1 kHz, and two grey broken lines show the average high and low time domain signals corresponding to the unblocked and blocked modes of the beam (by means of the optical chopper), respectively, indicating confocal reflectance. Doppler fluctuation can be seen on the high level of the time domain signal when the beam is not blocked.

The Doppler flowmetry and confocal reflectance parts of the LFI signal can be extracted from both the frequency (fast Fourier transformation of time domain signal) or the time domain signals. In order to show how the signal is processed to get both parts of the data out of it, frequency and time domain signals, acquired at a typical point inside the flow area, are shown in (a) and (b) of Figure 8, respectively. The red broken and black solid lines in (a) are the filtered Doppler spectrum (Savitzky–Golay filter) and the noise floor, respectively. Doppler flowmetry and confocal reflectance signals were calculated in the frequency domain by applying the filtered Doppler spectrum to Equation (2) and measuring the magnitude of the first harmonic (as the signal was modulated at 1 kHz) with respect to the noise floor, respectively. On the other hand, one can look at the level of fluctuations in the time domain (when the beam is not blocked by the chopper) and the difference between the average low and high levels of modulated signal, corresponding to the blocked and unblocked modes of the beam, to extract Doppler flowmetry and confocal reflectance signals. Figure 8 shows all of the aforementioned annotations. As can be seen in Figure 8, the Doppler spectrum is a broadened spectrum around the frequency of zero, because the beam in the suggested confocal LFI apparatus is normal to the flow. This configuration in conjunction with a high numerical aperture objective lens resulted in a broadened spectrum that was used in this work to extract Doppler flowmetry or the perfusion signal.

## 5. Discussion

We investigated and characterized the proposed imaging technique by applying it numerically to two typical types of tumourous skin as mild and severe KSCs (KSC1 and KSC2) in this work. We derived the optical properties of KSCs from the literature and take into account the lowest level of changes in the optical scattering and absorption coefficients of the tumours in our simulations [8,23], in order to focus on the improvement in the sensitivity of the proposed technique. Malignant melanomas, which is not included in the numerical models in this article, have different shapes, relative locations in the skin tissue, alterations in optical properties, etc., compared to those of KSCs. It has been reported that total reflectance is generally reduced with the increase in the degree of malignancy in the pigmented skin lesions in wide spectral ranges [67,68,69,70,71]. As can be seen in Figure 3, contrast in the multiplication images increases with the disease in severity, from KSC1 to KSC2. The increase in the scattering and absorption coefficients of malignant melanoma with respect to common nevi has also been reported [72,73]. A reason for the increase of the scattering coefficient with malignancy in skin lesions could be the increase in nuclear to cell volume with the degree of malignancy in tumourous cells [74]; as in the case of cervical carcinoma, the increase in nuclear to cell volume is a well-known cytological indicator of the tumour malignancy [75].

**Figure 9:**
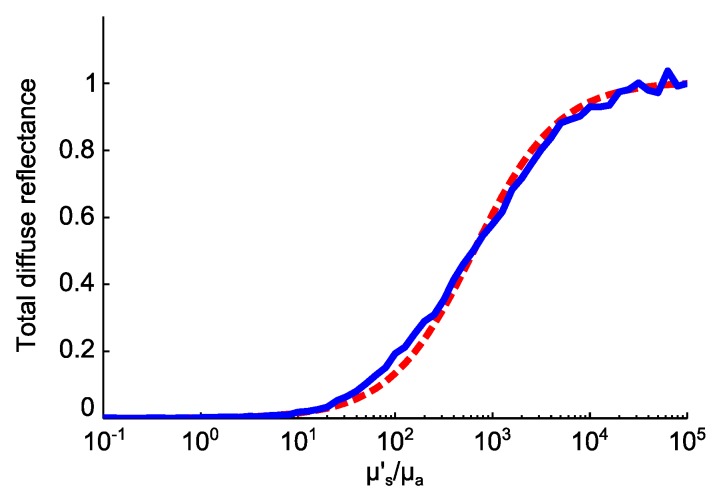
Simulated total reflectance from a typical tumourous skin model in our model (blue line) compared to the diffusion theory formula (red broken line), versus the ratio of the reduced scattering coefficient to absorption coefficient.

The reduction in total reflectance can be attributed to a decrease in the ratio of $\mu_{s}^{\prime }/\mu_{a}$ of the tumourous tissue. This means that higher absorption or lower scattering coefficients in skin tissue are expected to reduce the total reflectance. Therefore, the total reflectance level is more properly determined by considering the combination of optical properties. The total reflectance approximation formula derived from diffusion theory for a semi-infinite turbid medium has the following form [69]:

(5) $$R_{p}=\frac{1}{k_{1}\frac{1}{\mu_{s}^{\prime }}+k_{2}\frac{\mu_{a}}{\mu_{s}^{\prime }}}\;,$$

where $R_{p}$ is the detected portion of the total reflectance, $\mu_{a}$ and $\mu_{s}^{\prime }$ are the absorption and reduced scattering coefficients, respectively, and $k_{1}$ and $k_{2}$ are empirical parameters coming from the optical geometry of the setup. Figure 9 shows a comparison between the total reflectance against $\mu_{s}^{\prime }/\mu_{a}$, from Equation (5) and our Monte Carlo skin model, described in Subsection 2.3. As can be seen, there is good agreement between the analytic formula and the Monte Carlo simulation. We assumed $k_{1}$ and $k_{2}$ of the optical system model to be three and one, respectively, and the anisotropy factor to be 0.85 in the analytic formula. The Monte Carlo simulation in Figure 9 is performed by keeping the absorption coefficients of each layer as they are and sweeping the reduced scattering coefficients. We assumed an anisotropy factor of 0.85 in the Monte Carlo simulation, as well.

We defined an SBR parameter as a touchstone to quantify the improvement in the sensitivity of the technique. Both simulation and experimental results concluded that combining two modalities increases the sensitivity dramatically. Numerical SBR values presented in Section 3 show that the ratios of multiplication SBRs to Doppler flowmetry SBRs increase by factors of 2.0 and 1.7, and the ratios of multiplication SBRs to confocal reflectance SBRs increase by factors of 2.2 and 4.2 for typical KSC1 and KSC2, respectively. In a similar way, experimental SBR values presented in Subsection 4.2 show that the ratio of multiplication SBR to Doppler flowmetry and confocal reflectance SBRs increase by factors of 1.8 and 3.7 for the microfluidic setup, respectively. Depending on the concentration of the dynamic turbid medium used in the experiment, SBRs have similar trends as the values obtained from numerical models, which shows the consistency between simulations and experiment.

Skin neoplastic diseases often develop from pre-existing skin lesions [76]. Actinic keratoses and dysplastic nevi are the most important precursors to SCCs and melanomas, respectively [76]. The sizes of actinic keratosis lesions are usually 1–3 mm, but can be up to several centimetres [77], as well, and the sizes of dysplastic nevi are mostly larger than 5 mm [76]. Hence, we assumed that the sizes of the tumourous tissues are 5 mm in diameter in our numerical models. BCC and SCC are different histopathologically, and the origin cells of sporadic BCCs are less well defined; there are not any well-known precursors to them [78].

Our assumptions in defining the geometry, location and properties of tumour models include generalization to make the study of the proposed technique easier. For instance, seborrhoeic keratosis, as probably the most common benign KSC, is different from SCC and BCC histopathologically. It usually occurs just as a thickened epidermis and no extension into the dermis layer, but still may rarely mimic KSC or MM clinically. Moreover, KSC can be thought of in the context of a field cancerization model. Therefore, care should be taken in determining where the healthy tissue is. Field cancerization theory states that there is a genomically-unstable area around the primary tumour, inclined to aberrant growth [79,80]. Neovascularization as an abnormal growth requirement happens mainly in the primary neoplastic tissue. Therefore, the area around the primary tumour is called healthy tissue in this paper. In melanoma, the suspect lesion is pigmented, and it is easier to distinguish the perilesional skin area.

Some obstacles to early skin cancer detection are the large skin surface area and its susceptibility to other types of skin lesions that can resemble a type of skin cancer, which are hard to differentiate. For example, differentiating between melanoma and dysplastic nevi is challenging clinically and histopathologically [81]. Optical properties [71] and perfusion [24] of dysplastic nevi have been studied, and it is shown that melanoma has higher perfusion and lower reflectance compared to dysplastic nevi. We are including both of these modalities in our method, and this combination can increase the specificity of detection. A higher concentration of RBCs changes the optical properties of the skin, as blood has a high scattering coefficient, and the out-flow of fluid and cells from the walls of leaky newly-developed venules [20] is another reason for the distorted optical properties. Examining the morphology and flow direction of these newly-developed venules also has the potential of discriminating between non-pigmented neoplastic skin lesions and assessing their degree of malignancy [82].

Our proposed technique implemented in a hardware platform would afford a non-invasive, in vivo and non-contact skin investigation tool with high spatial resolution (as the focused beam in the experiment has a $1/e^{2}$ power beam spot diameter of about 1.8 µm). Optical properties of human skin can vary greatly in different places of the body and between individuals, and it will affect both the confocal reflectance and Doppler flowmetry signals greatly. One advantage of our technique is that it focuses on changes relative to surrounding tissue. Therefore, there is no need for calibration or measurement of absolute values. In addition to early detection, this technique could also be useful in differentiating different types of skin tumours by quantifying the level of neovascularization and optical property distortions, estimating the stage of tumourous development and the rate of growth by monitoring and delineating a tumour’s border for proper removal surgery [83].

Furthermore, care should be taken about the safe level of the laser beam optical power, and power should be limited by factors, such as the hazard of tissue damage and skin sensitivity [84]. Most of the commercially available laser scanning confocal microscopes use in-plane laser diodes with tens of mW of power [85] to achieve a signal to noise ratio that is detectable by the photodetector in a direct detection scheme; while our scheme uses a heterodyne detection scheme, in which a low power reflected signal mixes with strong intra-cavity fields (fields within the VCSEL’s cavity). In such a system, the sensitivity is so high, that the signal is detectable when laser operates at currents just above the threshold, and the output power is less than 100 µW. Such a low power system is not only safe, but also has the minimum effect on the optical properties and the structure of biological tissues.

## 6. Conclusions

Cancer is the result of complex interactions between environmental and genetic factors. Cancer incidence is growing each year, and early detection can save many lives and reduce the burden of disease. One way to develop a robust early detection method is to include all of the possible potential cancer indicators in the detection system. We suggest modulating the laser beam in a confocal LFI system to measure confocal reflectance at the same time as it measures the Doppler flowmetry signal. This results in a new imaging technique of simultaneous probing for two abnormal cancer biological traits of distorted tissue optical properties and perfusion. It can improve the capabilities of the currently existing systems, which use only one of these modalities, through improving the sensitivity and specificity. We examined this technique through a range of numerical simulations and conducted an experiment to image a dynamic turbid medium. Simulated and experimental results showed a high degree of consistency, which validates the technique. We also quantified the results by defining an SBR parameter as the ratio of the signal from the tumourous (or dynamic turbid) area to the signal from the background area. The signal to background ratio of the combined images has increased remarkably in both simulated and experimental examinations with similar trends, which indicates the enhanced sensitivity of the technique.

## Figures and Tables

**Table 1:** Six-layer skin tissue model, data from [56].

Layers Top to Bottom	Epidermis	Papillary Dermis	Superior Blood Net	Reticular Dermis	Inferior Blood Net	Subcutis Subcutis
0.3-mm/s blood concentration (%)	0	0.2	0.6	0.1	0.25	0.1
3.0-mm/s blood concentration (%)	0	0	0.05	0.01	0.035	0.01
30-mm/s blood concentration (%)	0	0	0.001	0.0006	0.006	0.001
Melanin concentration (%)	2.0	0	0	0	0	0
Thickness (mm)	0.075	0.15	0.15	0.8	0.4	2
$\mu_{s}$ bloodless melanin-less (mm^−1^)	23	13	13	13	13	13
$\mu_{a}$ bloodless melanin-less (mm^−1^)	0.1	0.1	0.1	0.1	0.1	0.1
Refractive index	1.4	1.4	1.4	1.4	1.4	1.4

## Abbreviations

LFI	Laser feedback interferometry
RCM	Reflectance confocal microscopy
SBR	Signal to background ratio
KSC	Keratinocyte skin cancer
BCC	Basal cell carcinoma
SCC	Squamous cell carcinoma
LDF	Laser Doppler flowmetry
VCSEL	Vertical-cavity surface-emitting laser

## References

[1] Diepgen T., Mahler V. (2002). The epidemiology of skin cancer. Br. J. Dermatol..

[2] Lomas A., Leonardi-Bee J., Bath-Hextall F. (2012). A systematic review of worldwide incidence of nonmelanoma skin cancer. Br. J. Dermatol..

[3] Soyer H.P., Rigel D., Wurm E.M. (2012). Actinic Keratosis, Basal Cell Carcinoma and Squamous Cell Carcinoma. Dermatology.

[4] Baldi A., Pasquali P., Spugnini E.P. (2014). Skin Cancer: A Practical Approach.

[5] Walling H.W., Fosko S.W., Geraminejad P.A., Whitaker D.C., Arpey C.J. (2004). Aggressive basal cell carcinoma: presentation, pathogenesis, and management. Cancer Metastasis Rev..

[6] Miller S.J. (1991). Biology of basal cell carcinoma (Part I). J. Am. Acad. Dermatol..

[7] Anderson R.R., Parrish J.A. (1981). The optics of human skin. J. Investig. Dermatol..

[8] Salomatina E., Jiang B., Novak J., Yaroslavsky A.N. (2006). Optical properties of normal and cancerous human skin in the visible and near-infrared spectral range. J. Biomed. Opt..

[9] Zonios G., Perelman L.T., Backman V., Manoharan R., Fitzmaurice M., Van Dam J., Feld M.S. (1999). Diffuse reflectance spectroscopy of human adenomatous colon polyps in vivo. Appl. Opt..

[10] Zonios G., Bykowski J., Kollias N. (2001). Skin melanin, hemoglobin, and light scattering properties can be quantitatively assessed in vivo using diffuse reflectance spectroscopy. J. Investig. Dermatol..

[11] Webb R.H. (1996). Confocal optical microscopy. Rep. Prog. Phys..

[12] Rajadhyaksha M., Grossman M., Esterowitz D., Webb R.H., Anderson R.R. (1995). In vivo confocal scanning laser microscopy of human skin: Melanin provides strong contrast. J. Investig. Dermatol..

[13] Hofmann-Wellenhof R., Pellacani G., Malvehy J., Soyer H.P. (2012). Reflectance confocal microscopy for skin diseases.

[14] Longo C., Farnetani F., Ciardo S., Cesinaro A., Moscarella E., Ponti G., Zalaudek I., Argenziano G., Pellacani G. (2013). Is confocal microscopy a valuable tool in diagnosing nodular lesions? A study of 140 cases. Br. J. Dermatol..

[15] Scope A., Mahmood U., Gareau D., Kenkre M., Lieb J., Nehal K., Rajadhyaksha M. (2010). In vivo reflectance confocal microscopy of shave biopsy wounds: Feasibility of intraoperative mapping of cancer margins. Br. J. Dermatol..

[16] Rishpon A., Kim N., Scope A., Porges L., Oliviero M.C., Braun R.P., Marghoob A.A., Fox C.A., Rabinovitz H.S. (2009). Reflectance confocal microscopy criteria for squamous cell carcinomas and actinic keratoses. Arch. Dermatol..

[17] Segura S., Puig S., Carrera C., Palou J., Malvehy J. (2009). Development of a two-step method for the diagnosis of melanoma by reflectance confocal microscopy. J. Am. Acad. Dermatol..

[18] Hanahan D., Weinberg R.A. (2011). Hallmarks of cancer: The next generation. Cell.

[19] Vaupel P., Kallinowski F., Okunieff P. (1989). Blood flow, oxygen and nutrient supply, and metabolic microenvironment of human tumours: A review. Cancer Res..

[20] Jain R.K. (2008). Taming vessels to treat cancer. Sci. Am..

[21] Rajan V., Varghese B., van Leeuwen T.G., Steenbergen W. (2009). Review of methodological developments in laser Doppler flowmetry. Lasers Med. Sci..

[22] Pigott K., Hill S., Chaplin D., Saunders M. (1996). Microregional fluctuations in perfusion within human tumours detected using laser Doppler flowmetry. Radiother. Oncol..

[23] Stücker M., Horstmann I., Nüchel C., Röchling A., Hoffmann K., Altmeyer P. (1999). Blood flow compared in benign melanocytic naevi, malignant melanomas and basal cell carcinomas. Clin. Exp. Dermatol..

[24] Stücker M., Esser M., Hoffmann M., Memmel U., Ller A.H., Von Bormann C., Hoffmann K., Altmeyer P. (2002). High-resolution laser Doppler perfusion imaging aids in differentiating between benign and malignant melanocytic skin tumours. Acta Derm Venereol.

[25] Wang I., Anderson-Engels S., Nilsson G., Wardell K., Svanberg K. (1997). Superficial blood flow following photodynamic therapy of malignant non–melanoma skin tumours measured by laser Doppler perfusion imaging. Br. J. Dermatol..

[26] Saravanamuthu J., Seifalian A., Reid W., Maclean A. (2003). A new technique to map vulva microcirculation using laser Doppler perfusion imager. Int. J. Gynecol. Cancer.

[27] Seifalian A., Chaloupka K., Parbhoo S. (1995). Laser Doppler perfusion imaging–a new technique for measuring breast skin blood flow. Int. J. Microcirc..

[28] Wårdell K., Jakobsson A., Nilsson G.E. (1993). Laser Doppler perfusion imaging by dynamic light scattering. IEEE Trans. Biomed. Eng..

[29] Chen Z., Milner T.E., Srinivas S., Wang X., Malekafzali A., van Gemert M.J., Nelson J.S. (1997). Noninvasive imaging of in vivo blood flow velocity using optical Doppler tomography. Opt. Lett..

[30] Yaroslavsky A.N., Barbosa J., Neel V., DiMarzio C., Anderson R.R. (2005). Combining multispectral polarized light imaging and confocal microscopy for localization of nonmelanoma skin cancer. J. Biomed. Opt..

[31] Giuliani G., Norgia M., Donati S., Bosch T. (2002). Laser diode self-mixing technique for sensing applications. J. Opt. A Pure Appl. Opt..

[32] Donati S. (2012). Developing self-mixing interferometry for instrumentation and measurements. Laser Photonics Rev..

[33] Donati S., Norgia M. (2014). Self-mixing interferometry for biomedical signals sensing. IEEE J. Sel. Top. Quantum Electron..

[34] Taimre T., Nikolić M., Bertling K., Lim Y.L., Bosch T., Rakić A.D. (2015). Laser Feedback Interferometry: A Guide to the Self-Mixing Effect for Coherent Sensing. Adv. Opt. Photonics.

[35] Perchoux J., Quotb A., Atashkhooei R., Azcona F.J., Ramírez-Miquet E.E., Bernal O., Jha A., Luna-Arriaga A., Yanez C., Caum J. (2016). Current Developments on Optical Feedback Interferometry as an All-Optical Sensor for Biomedical Applications. Sensors.

[36] Bearden A., O’Neill M.P., Osborne L.C., Wong T.L. (1993). Imaging and vibrational analysis with laser-feedback interferometry. Opt. Lett..

[37] Lu C.H., Wang J., Deng K.L. (1995). Imaging and profiling surface microstructures with noninterferometric confocal laser feedback. Appl. Phys. Lett..

[38] Lim Y.L., Kliese R., Bertling K., Tanimizu K., Jacobs P., Rakic A.D. (2010). Self-mixing flow sensor using a monolithic VCSEL array with parallel readout. Opt. Express.

[39] Campagnolo L., Nikolić M., Perchoux J., Lim Y.L., Bertling K., Loubiere K., Prat L., Rakić A.D., Bosch T. (2013). Flow profile measurement in microchannel using the optical feedback interferometry sensing technique. Microfluid. Nanofluid..

[40] Mowla A., Nikolic M., Lim Y.L., Bertling K., Rakic A.D., Taimre T. Effect of the optical numerical aperture on the Doppler spectrum in laser Doppler velocimetry. Proceedings of the 2014 IEEE Conference onOptoelectronic and Microelectronic Materials & Devices.

[41] Mowla A., Nikolić M., Taimre T., Tucker J.R., Lim Y.L., Bertling K., Rakić A.D. (2015). Effect of the optical system on the Doppler spectrum in laser-feedback interferometry. Appl. Opt..

[42] White J. (2014). Reflecting on confocal microscopy: A personal perspective. Confocal Microsc. Methods Protoc..

[43] Minsky M. (1988). Memoir on inventing the confocal scanning microscope. Scanning.

[44] White J., Amos W., Fordham M. (1987). An evaluation of confocal versus conventional imaging of biological structures by fluorescence light microscopy. J. Cell Biol..

[45] Juškaitis R., Rea N., Wilson T. (1994). Semiconductor laser confocal microscopy. Appl. Opt..

[46] Wang M., Lai G. (2004). Self-mixing microscopic interferometer for the measurement of microprofile. Opt. Commun..

[47] Tan Y., Wang W., Xu C., Zhang S. (2013). Laser confocal feedback tomography and nano-step height measurement. Sci. Rep..

[48] Mowla A., Taimre T., Lim Y.L., Bertling K., Wilson S.J., Prow T.W., Soyer H.P., Rakić A.D. (2016). Diffuse reflectance imaging for non-melanoma skin cancer detection using laser feedback interferometry. Proc. SPIE.

[49] Michalzik R. (2012). VCSELs: Fundamentals, Technology and Applications of Vertical-Cavity Surface-Emitting Lasers.

[50] Albrecht H.E. (2003). Laser Doppler and Phase Doppler Measurement Techniques.

[51] Figueiras E., Oliveira R., Lourenço C.F., Campos R., Humeau-Heurtier A., Barbosa R.M., Laranjinha J., Ferreira L.F.R., de Mul F.F. (2013). Self-mixing microprobe for monitoring microvascular perfusion in rat brain. Med. Biol. Eng. Comput..

[52] Wang L., Jacques S.L., Zheng L. (1995). MCML–Monte Carlo modeling of light transport in multi-layered tissues. Comput. Methods Progr. Biomed..

[53] Tuchin V. (2007). Tissue Optics: Light Scattering Methods and Instruments for Medical Diagnosis.

[54] Kroese D.P., Taimre T., Botev Z.I. (2013). Handbook of Monte Carlo Methods.

[55] Tycho A., Jørgensen T.M., Yura H.T., Andersen P.E. (2002). Derivation of a Monte Carlo method for modeling heterodyne detection in optical coherence tomography systems. Appl. Opt..

[56] Fredriksson I., Larsson M., Strömberg T. (2008). Optical microcirculatory skin model: Assessed by Monte Carlo simulations paired with in vivo laser Doppler flowmetry. J. Biomed. Opt..

[57] Hale G.M., Querry M.R. (1973). Optical constants of water in the 200-nm to 200-μm wavelength region. Appl. Opt..

[58] Hammer M., Yaroslavsky A.N., Schweitzer D. (2001). A scattering phase function for blood with physiological haematocrit. Phys. Med. Biol..

[59] Feng X., Patel R., Yaroslavsky A.N. (2015). Wavelength optimized cross-polarized wide-field imaging for noninvasive and rapid evaluation of dermal structures. J. Biophotonics.

[60] Jacques S.L. (1998). Skin optics. Or. Med. Laser Center News.

[61] Mowla A., Taimre T., Lim Y.L., Bertling K., Wilson S.J., Prow T.W., Rakić A.D. (2016). A Compact Laser Imaging System for Concurrent Reflectance Confocal Microscopy and Laser Doppler Flowmetry. IEEE Photonics J..

[62] De Mul F., Koelink M., Weijers A., Greve J., Aarnoudse J., Graaff R., Dassel A. (1992). Self-mixing laser-Doppler velocimetry of liquid flow and of blood perfusion in tissue. Appl. Opt..

[63] Matharu R.S., Perchoux J., Kliese R., Lim Y.L., Rakić A.D. (2011). Maintaining maximum signal-to-noise ratio in uncooled vertical-cavity surface-emitting laser-based self-mixing sensors. Opt. Lett..

[64] Flock S.T., Jacques S.L., Wilson B.C., Star W.M., van Gemert M.J. (1992). Optical properties of Intralipid: A phantom medium for light propagation studies. Lasers Surg. Med..

[65] Cubeddu R., Pifferi A., Taroni P., Torricelli A., Valentini G. (1997). A solid tissue phantom for photon migration studies. Phys. Med. Biol..

[66] Bertling K., Taimre T., Agnew G., Lim Y.L., Dean P., Indjin D., Höfling S., Weih R., Kamp M., von Edlinger M. (2016). Simple electrical modulation scheme for laser feedback imaging. IEEE Sens. J..

[67] Marchesini R., Brambilla M., Clemente C., Maniezzo M., Sichirollo A.E., Testori A., Venturoli D.R., Cascinelli N. (1991). In vivo spectrophotometric evaluation of neoplastic and non-neoplastic skin pigmented lesions–I. reflectance measurements. Photochem. Photobiol..

[68] Tomatis S., Bartoli C., Bono A., Cascinelli N., Clemente C., Marchesini R. (1998). Spectrophotometric imaging of cutaneous pigmented lesions: Discriminant analysis, optical properties and histological characteristics. J. Photochem. Photobiol. B Biol..

[69] Zonios G., Dimou A. (2006). Modeling diffuse reflectance from semi-infinite turbid media: Application to the study of skin optical properties. Opt. Express.

[70] Zonios G., Dimou A., Bassukas I., Galaris D., Tsolakidis A., Kaxiras E. (2008). Melanin absorption spectroscopy: New method for noninvasive skin investigation and melanoma detection. J. Biomed. Opt..

[71] Zonios G., Dimou A., Carrara M., Marchesini R. (2010). In vivo optical properties of melanocytic skin lesions: Common nevi, dysplastic nevi and malignant melanoma. Photochem. Photobiol..

[72] Garcia-Uribe A., Smith E.B., Zou J., Duvic M., Prieto V., Wang L.V. (2011). In-vivo characterization of optical properties of pigmented skin lesions including melanoma using oblique incidence diffuse reflectance spectrometry. J. Biomed. Opt..

[73] Garcia-Uribe A., Zou J., Duvic M., Cho-Vega J.H., Prieto V.G., Wang L.V. (2012). In vivo diagnosis of melanoma and nonmelanoma skin cancer using oblique incidence diffuse reflectance spectrometry. Cancer Res..

[74] Jorgensen P., Edgington N.P., Schneider B.L., Rupeš I., Tyers M., Futcher B. (2007). The size of the nucleus increases as yeast cells grow. Mol. Biol. Cell.

[75] Slater D., Rice S., Stewart R., Melling S., Hewer E., Smith J. (2005). Proposed Sheffield quantitative criteria in cervical cytology to assist the grading of squamous cell dyskaryosis, as the British Society for Clinical Cytology definitions require amendment. Cytopathology.

[76] Sober A.J., Burstein J.M. (1995). Precursors to skin cancer. Cancer.

[77] Schwartz R., Bridges T., Butani A., Ehrlich A. (2008). Actinic keratosis: An occupational and environmental disorder. J. Eur. Acad. Dermatol. Venereol..

[78] Lacour J. (2002). Carcinogenesis of basal cell carcinomas: Genetics and molecular mechanisms. Br. J. Dermatol..

[79] Slaughter D.P., Southwick H.W., Smejkal W. (1953). Field cancerization in oral stratified squamous epithelium. Clinical implications of multicentric origin. Cancer.

[80] Kanjilal S., Strom S.S., Clayman G.L., Weber R.S., El-Naggar A.K., Kapur V., Cummings K.K., Hill L.A., Spitz M.R., Kripke M.L. (1995). p53 mutations in nonmelanoma skin cancer of the head and neck: Molecular evidence for field cancerization. Cancer Res..

[81] Elbaum M., Kopf A.W., Rabinovitz H.S., Langley R.G., Kamino H., Mihm M.C., Sober A.J., Peck G.L., Bogdan A., Gutkowicz-Krusin D. (2001). Automatic differentiation of melanoma from melanocytic nevi with multispectral digital dermoscopy: A feasibility study. J. Am. Acad. Dermatol..

[82] Incel P., Gurel M., Erdemir A. (2015). Vascular patterns of non-pigmented tumoural skin lesions: Confocal perspectives. Skin Res. Technol..

[83] Yaroslavsky A.N., Neel V., Anderson R.R. (2003). Demarcation of nonmelanoma skin cancer margins in thick excisions using multispectral polarized light imaging. J. Investig. Dermatol..

[84] Marks R., Edwards C. (1992). The measurement of photodamage. Br. J. Dermatol..

[85] Calzavara-Pinton P., Longo C., Venturini M., Sala R., Pellacani G. (2008). Reflectance confocal microscopy for in vivo skin imaging. Photochem. Photobiol..

